# The p21 resilience network: a conceptual framework linking senescence to ferroptosis and cuproptosis resistance

**DOI:** 10.1038/s41540-026-00682-7

**Published:** 2026-03-09

**Authors:** Shantanu Gupta

**Affiliations:** Bioinformatics Multidisciplinary Environment-BioME—Digital Metropole Institute, Federal University of Rio Grande do Norte, Natal, RN Brazil

**Keywords:** Cancer, Cell biology, Oncology

## Abstract

We reconceptualize p21 as the master regulator of a “resilience network” that empowers cancer cells to defy therapy. Beyond cell cycle arrest, p21 orchestrates a multi-faceted defense, suppressing ferroptosis and cuproptosis by governing redox and metal ion homeostasis. This perspective demands a therapeutic paradigm shift: targeting the p21 network is essential to dismantle this evolved survival machinery and force durable tumor regression in resistant cancers.

## Introduction

The cyclin-dependent kinase inhibitor p21^^CDKN1A^ is one of the most extensively studied downstream effectors of the tumor suppressor p53^1^. Historically, p21 was defined as a canonical regulator of cell cycle arrest, primarily functioning as a safeguard by inhibiting Cyclin-dependent kinase (CDK) complexes and enforcing G1/S and G2/M checkpoints following DNA damage^2^. This protective pause enables repair mechanisms to restore genomic integrity before proliferation resumes.

Over the last two decades, however, p21 has emerged as much more than a passive checkpoint regulator, with its stability and function being governed by complex post-transcriptional mechanisms^3^. Its biological repertoire spans the induction of senescence, modulation of DNA repair pathways, context-dependent regulation of apoptosis^4^ and autophagy^5^, and as explored in this Perspective, resistance to non-apoptotic cell death programs. Rather than acting as a linear executor of p53 signaling, p21 functions as a molecular switch, integrating stress signals and orchestrating divergent outcomes: reversible arrest, permanent senescence, or resistance against multiple forms of programmed cell death.

The recent discovery of novel Regulated cell death (RCD) pathways, such as ferroptosis^6^ and cuproptosis^7^, has expanded our understanding of cell fate control. Ferroptosis is an iron-catalyzed form of death characterized by the lethal accumulation of phospholipid hydroperoxides^6^, while cuproptosis is triggered by excessive copper that leads to the aggregation of mitochondrial enzymes and proteotoxic stress^7^. These pathways represent promising avenues for cancer therapy, but their efficacy is limited by inherent resistance mechanisms.

The tumor suppressor p53 has long been described as a master regulator of cell fate^8^, with an established ability to either induce or suppress ferroptosis through distinct transcriptional programs^9–11^. Many studies have focused on this dual role of p53, particularly highlighting how p21 acts as a downstream effector mediating ferroptotic resistance^9,10^. However, the centrality of p21 itself has often been underappreciated. Most existing reviews describe ferroptotic regulation through the lens of p53, treating p21 as an auxiliary factor. In this Perspective, we shift the focus explicitly toward p21, arguing that it functions not merely as a downstream extension of p53 but as an autonomous and convergent inhibitor of regulated cell death. This focus on apoptosis, autophagy, ferroptosis, and cuproptosis is deliberate, as these pathways have the strongest direct evidence linking p21 to them. While the RCD spectrum is broad (encompassing necroptosis^12,13^, pyroptosis^14^, and others), these additional pathways are beyond the scope of this perspective. We synthesize evidence positioning p21 as a nodal survival regulator and a unifying barrier against them, with ferroptosis and cuproptosis being intriguingly linked through the shared p21- Nuclear factor erythroid 2-related factor 2 (Nrf2)- Glutathione (GSH) axis. These pathways represent the most well-established and functionally coherent components of the ‘p21 resilience network’.

This duality makes p21 an ambivalent player in cancer biology^15^. While transient activation can protect genomic stability, prolonged or context-dependent expression may confer survival advantages to malignant cells under therapy-induced stress. Here, we develop the concept of p21 as a multidimensional survival factor and introduce the “p21 resilience network.” We integrate experimental evidence to support the argument that p21 operates as a unifying regulator across classical and novel death pathways, outlining a new framework for understanding and targeting therapy resistance.

## p21 in cell cycle arrest and DNA repair

The most classical role of p21 is in halting cell cycle progression following DNA damage. Activated predominantly by p53, p21 binds cyclin–CDK complexes, including cyclin E–CDK2 and cyclin A–CDK2 at the G1/S transition, as well as cyclin B–CDK1 at G2/M^4,16^. This inhibition prevents cells carrying damaged DNA from advancing into replication or mitosis, thereby preserving genomic integrity.

Beyond checkpoint enforcement, p21 directly influences DNA repair mechanisms. Through its interaction with proliferating cell nuclear antigen (PCNA), p21 regulates nucleotide excision repair and base excision repair pathways^17^. This interaction fine-tunes replication fork progression and coordinates DNA repair machinery with cell cycle arrest, ensuring a seamless coupling between damage sensing and genome maintenance^18^.

Experimental studies reinforce this dual role. Knockout of p21 accelerates replication fork collapse and chromosomal aberrations following genotoxic stress, whereas enforced expression delays replication but allows more efficient lesion repair^19^. Such findings highlight that transient p21 induction favors cell survival by “buying time” for repair, whereas sustained activation may drive cells into senescence or confer therapy resistance depending on context.

## p21 in senescence and context-dependent outcomes

Senescence represents an irreversible growth arrest often triggered by persistent DNA damage, telomere attrition, or oncogene activation^20^. p21 is among the earliest mediators of this process, acting downstream of both p53-dependent and -independent pathways^21^. Persistent p21 expression maintains CDK inhibition, enforcing long-term arrest.

In oncogene-induced senescence (OIS), p21 collaborates with p16^^INK4a^ to provide a tumor-suppressive barrier^22^. Loss of this barrier allows cells to bypass oncogenic stress, accelerating tumorigenesis. Yet senescence is a double-edged sword^23,24^: while p21-induced senescence initially halts malignant progression, senescent cells later secrete a senescence-associated secretory phenotype (SASP) rich in cytokines and proteases^25^. This can stimulate immune clearance but also foster chronic inflammation and tumor progression^26^.

Experimental models demonstrate this duality. Mouse models with enforced p21 expression develop strong senescence responses and reduced early tumor burden, yet chronic accumulation of senescent cells accelerates tissue dysfunction and supports late-stage tumor growth^27^. This is further supported by Galanos et al.^28^, who demonstrated for the first time that chronic p21 expression leads to escape from senescence by enhancing genomic instability and deregulating replication licensing^28^. Thus, p21-driven senescence is protective in acute settings but deleterious when persistent, underscoring its role as a context-dependent regulator rather than a simple tumor suppressor.

## p21 as an inhibitor of apoptosis

Another layer of p21’s complex biology is its capacity, in certain contexts, to blunt apoptosis, thereby tipping stressed cells toward survival. p21 exerts this anti-apoptotic function through several key mechanisms, predominantly when localized in the cytoplasm. It can directly bind and inhibit initiator and effector caspases, such as caspase-3 and caspase-8, thereby blocking the execution phase of apoptosis^15,29^. Furthermore, cytoplasmic p21 interacts with apoptosis signal-regulating kinase 1 (ASK1), dampening stress kinase–driven apoptotic cascades^30,31^.

Experimental evidence highlights this critical anti-apoptotic role. In p21-deficient tumor cells exposed to DNA-damaging agents, apoptosis occurs rapidly, whereas cells with enforced p21 expression survive despite accumulating damage^32^. Similarly, the cytoplasmic localization of p21 has been correlated with chemoresistance in colorectal^33^ and breast cancer^34^, consistent with its role in suppressing cell death.

These data illustrate a key paradox: p21, once heralded as a tumor suppressor, can become a protector of malignant cells under therapy pressure. Its ability to simultaneously induce senescence and block apoptosis enables resistant subclones to survive cytotoxic therapies, consolidating its function as a molecular shield.

## p21 in autophagy regulation

Autophagy, the self-digestion process that recycles cytoplasmic components, plays ambivalent roles in cancer, and p21’s regulation of it is equally complex and context-dependent. p21 has been demonstrated to have dual roles, capable of both promoting and suppressing autophagy, with the outcome largely determined by its subcellular localization and the cellular context.

- Pro-autophagic Role: In its nuclear, tumor-suppressive capacity, p21 can promote autophagic cell death as part of a coordinated stress response. This is achieved through the transcriptional upregulation of key autophagy initiators, including ULK1 and LC3, thereby activating a pro-death program^5^.
- Anti-autophagic Role: In contrast, and more relevant to the development of therapy resistance, p21 is a potent suppressor of autophagic flux. This function is frequently linked to its cytoplasmic localization. Mechanistically, emerging evidence demonstrates that p21 acts as a crucial negative regulator of the Akt signaling axis. p21 deficiency leads to constitutive Akt activation, which in turn drives ROS accumulation through the FoxO-dependent downregulation of antioxidant genes. This sustained ROS elevation ultimately functions as a potent inducer of autophagy^35^. Therefore, physiological p21 serves to restrain this Akt-ROS cascade, thereby maintaining low basal autophagy. Experimental studies demonstrate that p21-deficient cells show elevated autophagy following nutrient stress, whereas its overexpression blocks autophagic vesicle formation and enhances survival under starvation^36,37^.

The significance of this suppression becomes clear in therapeutic settings. Apoptosis-resistant tumors often rely on autophagy as a backup death mechanism^38^. By concurrently suppressing both apoptosis^4^ and autophagic flux^35^, p21 consolidates its role as a master gatekeeper of cellular survival. Cells with persistent p21 can thereby withstand metabolic and genotoxic stresses that would otherwise induce cell death^20^, consolidating its function within the anti-tumorigenic ‘p21 resilience network’.

## p21 in ferroptosis: experimental evidence and mechanistic insights

Ferroptosis, an iron-dependent form of regulated cell death driven by lipid peroxidation, has emerged as a key mechanism in cancer biology and degenerative disease^6^. While p53 has been widely discussed as both an inducer and suppressor of ferroptosis^9–11^, often through the transcriptional induction of p21, comparatively few studies have examined p21 as a direct regulator. Here, we argue that p21 is not simply a mediator of p53’s anti-ferroptotic function but rather a pivotal inhibitor in its own right. This reframing distinguishes p21 as a molecular barrier that independently governs ferroptotic sensitivity, positioning it as a survival “switch” analogous to its roles in apoptosis and autophagy.

A landmark study by Venkatesh et al. established p21 as a central molecular barrier to ferroptosis, providing a continuum of evidence from correlation to mechanism^39^. They demonstrated that basal p21 expression directly correlates with ferroptotic sensitivity: p21-high cancer cell lines are resistant to inducers like erastin and RSL3, whereas p21-low lines are highly sensitive. This phenotype was functionally reversible; knockdown of p21 sensitized resistant lines, while its enforced expression protected sensitive ones. While these phenotypes are consistent with ferroptosis using standard inducers, comprehensive validation (e.g., ferrostatin-1 rescue, lipid peroxidation measurements, or genetic knockdown) would further strengthen this phenomenon. Crucially, this regulatory role persisted in p53-deficient contexts, confirming p21’s autonomous function^39^.

Intriguingly, p21 itself is dynamically regulated during ferroptotic stress, functioning as a stress-responsive node. The same study^39^ revealed that in resistant cells, p21 protein levels are sustained or even induced upon treatment, while they are depleted in sensitive cells - a pattern observed irrespective of p53 status. This suggests a selective post-transcriptional mechanism that stabilizes p21 specifically in cells poised to survive, reinforcing its role in an active pro-survival feedback loop^39^.

Mechanistically, p21 suppresses ferroptosis through multiple, non-mutually exclusive pathways. A primary mechanism involves stabilizing glutathione peroxidase 4 (GPX4), a master inhibitor of lipid peroxidation. p21 loss destabilizes GPX4, increasing lipid ROS and ferroptotic death. Importantly, this occurs independently of Nrf2, relying instead on regulation of GPX4 ubiquitination via the linear ubiquitin chain assembly complex (LUBAC)^39^ (proposed mechanism based on converging evidence). Furthermore, emerging evidence reveals a parallel pathway where p21 directly interacts with and activates the transcription factor Nrf2, a master regulator of the antioxidant response^40^. This p21/Nrf2 axis has been demonstrated to be a critical inhibitor of ferroptosis, and notably, this activation can occur through both p53-dependent and p53-independent mechanisms^40^. While p21–Nrf2 interaction is demonstrated, loss-of-function epistasis experiments would be needed to establish necessity versus association.

The physiological relevance of this network is highlighted by studies in osteoarthritis chondrocytes, where p21 knockdown decreased GPX4 and sensitized cells to ferroptosis^41^, confirming its role beyond cancer.

Together, these findings establish p21 as a ferroptosis suppressor across diverse systems, from cancer to degenerative disease. Therapeutically, this means that p21-targeted interventions could unlock vulnerability to ferroptosis in otherwise resistant cancers, providing a novel route to overcome apoptosis- and autophagy-resistant tumors.

## p21 and cuproptosis: a unified defense through the Nrf2-GSH axis

Cuproptosis, a copper-dependent regulated cell death pathway, is triggered by copper binding to lipoylated Tricarboxylic acid (cycle) (TCA) cycle proteins, resulting in proteotoxic stress and Reactive oxygen species (ROS) accumulation^7,42,43^. While mechanistically distinct from ferroptosis, a compelling hypothesis for p21-mediated cuproptosis resistance emerges from their shared reliance on GSH metabolism. Critically, recent experimental studies have solidified GSH’s role as a primary physiological inhibitor of cuproptosis, where it functions as a direct copper chelator to prevent mitochondrial copper accumulation^44–46^. The depletion of GSH is a proven and effective strategy to sensitize cells to cuproptosis^44,45^.

This places the well-established p21-Nrf2 axis in a new, pivotal context. We have previously outlined how p21 stabilizes Nrf2 to activate the antioxidant response^39,40,47^. A central outcome of Nrf2 activation is the transcriptional upregulation of genes responsible for GSH synthesis^46^. Therefore, by enhancing the cellular GSH pool, the p21-Nrf2 pathway is mechanistically equipped to buffer against cuproptosis. This model is reinforced by the finding that genetic inhibition of the NFE2L2 (Nrf2)-GSH axis sensitizes cells to cuproptosis, confirming its role as a core cellular defense pathway^46^.

**Fig. 1:**
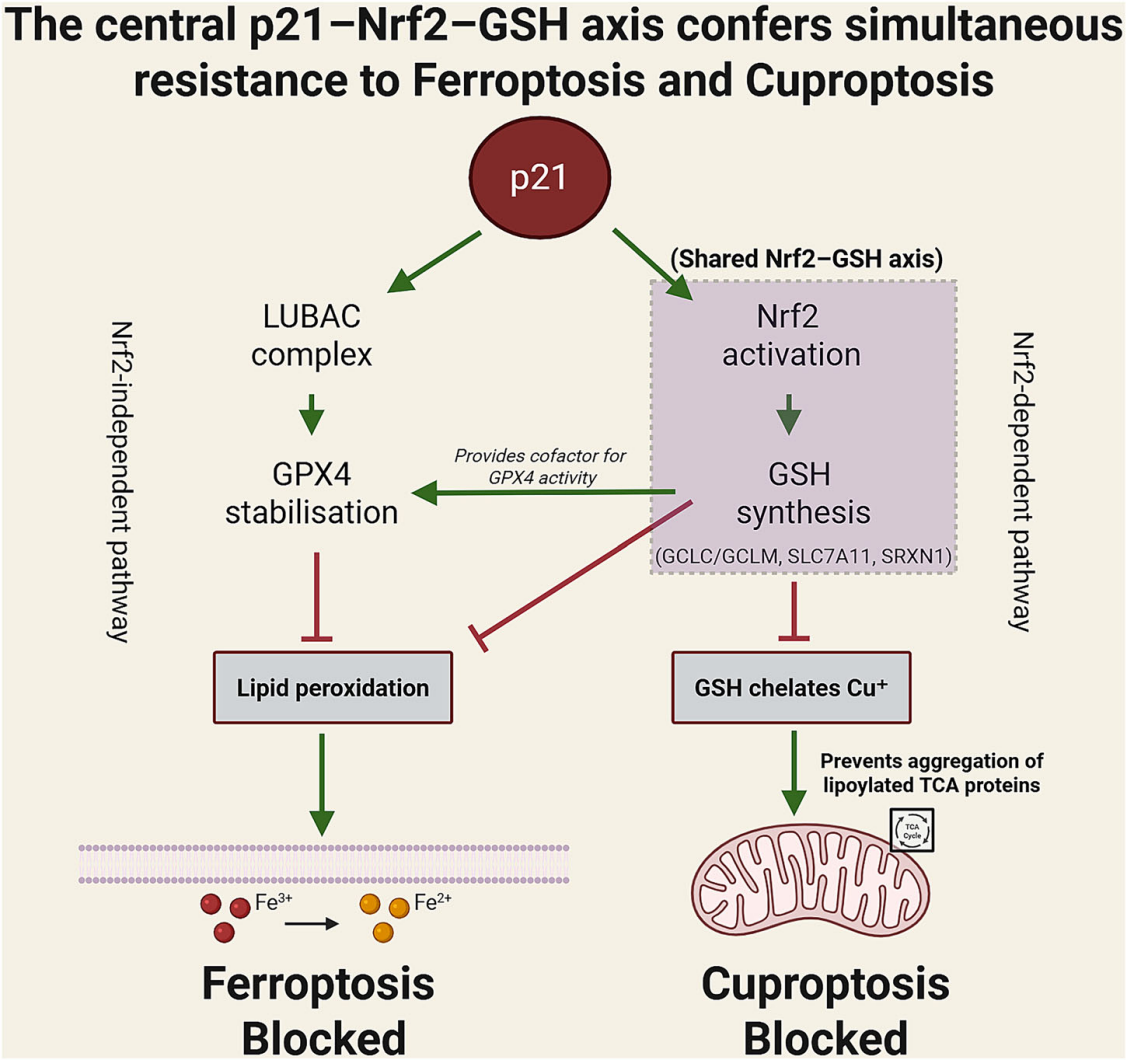
p21 orchestrates a unified Nrf2–GSH axis that simultaneously suppresses ferroptosis and cuproptosis. p21 orchestrates a unified resilience program via two parallel protective pathways. The left (Nrf2-independent pathway) stabilises GPX4 protein through the LUBAC complex. The right (Nrf2-dependent pathway, highlighted in purple) drives GSH synthesis (GCLC/GCLM, SLC7A11, SRXN1). Elevated GSH acts as the convergent downstream effector: it provides the essential cofactor for GPX4 catalytic activity (detoxifying lipid hydroperoxides and blocking ferroptosis) and directly chelates excess Cu^+^ (preventing aggregation of lipoylated TCA-cycle proteins and blocking cuproptosis). This shared p21–Nrf2–GSH axis (purple box) represents a novel conceptual model proposed in this perspective, suggesting a potential broad-spectrum resistance mechanism against metal-induced regulated cell death. Green arrows indicate activation; red hammer-head arrows indicate inhibition. Note: Solid lines/arrows represent experimentally established mechanisms supported by cited literature. The unified p21–Nrf2–GSH axis (purple box) and its proposed broad-spectrum role in suppressing both ferroptosis and cuproptosis represent a novel conceptual model introduced in this perspective.

This proposed mechanism aligns perfectly with p21’s established function as a metabolic rheostat and its pro-survival role in the context of ferroptosis. It suggests that the “p21 resilience network” defends against diverse metal-induced death pathways by orchestrating a common antioxidant and detoxification response, positioning p21 as a master regulator of metal ion homeostasis and a promising target to sensitize cells to cuproptosis-inducing therapies. Fig. 1 illustrates the proposed unified model in which a single p21-driven Nrf2–GSH axis simultaneously suppresses ferroptosis (via lipid-peroxide detoxification) and cuproptosis (via copper chelation and protection of lipoylated TCA-cycle proteins).

## Integrative model: p21 as a molecular switch

**Fig. 2:**
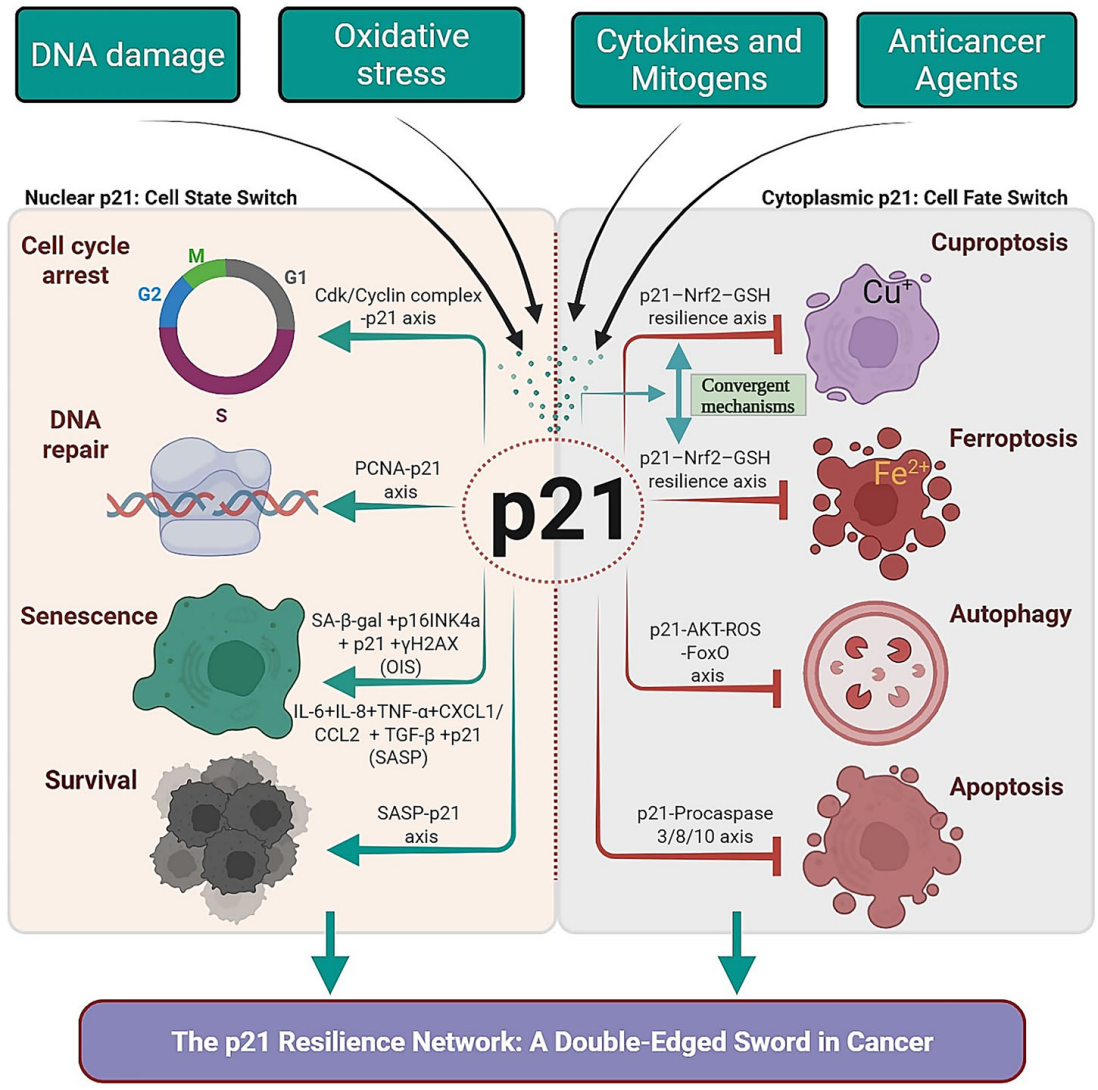
The p21 resilience network: a double-edged sword in cancer. p21 functions as a localisation-dependent molecular switch in response to diverse stresses (DNA damage, oxidative stress, cytokines, anticancer agents). Nuclear p21 (left, teal/green arrows = activation) drives cell-cycle arrest, DNA repair, and senescence. In contrast, cytoplasmic p21 (right, red hammer-head arrows = inhibition) simultaneously blocks multiple regulated cell death pathways—apoptosis, autophagy, ferroptosis, and cuproptosis—through convergent downstream mechanisms, with the shared p21–Nrf2–GSH resilience axis (highlighted in green and labeled “Convergent Mechanism”) representing a novel conceptual resistance mechanism proposed in this perspective. This dual action establishes the “p21 resilience network” that allows cells—particularly therapy-induced senescent cells—to persist and contribute to tumor relapse. Note: Solid lines/arrows (including black activation arrows from stresses to p21 and teal/green arrows for nuclear activation) and red hammer-head arrows represent experimentally established mechanisms supported by cited literature. The unified p21–Nrf2–GSH resilience axis (highlighted in green and labeled “Convergent Mechanism”) and its proposed role in broad-spectrum inhibition of ferroptosis and cuproptosis represent a novel conceptual model introduced in this perspective.

Synthesizing these insights, p21 emerges not as a single-function effector but as a molecular rheostat capable of tuning cell fate outcomes across multiple programs^48^. Short-term induction enforces cell cycle arrest and DNA repair. Persistent induction promotes senescence. Cytoplasmic localization inhibits apoptosis and autophagy. Furthermore, p21 orchestrates a centralized defense against metabolic cell death by activating the Nrf2 antioxidant pathway. This axis, by boosting GSH synthesis, concurrently shields cells from both ferroptosis (by mitigating lipid peroxidation) and cuproptosis (by chelating copper ions). This integrated role positions p21 as a critical determinant of therapeutic resistance. By concurrently blocking multiple death pathways—apoptosis, autophagy, ferroptosis, and cuproptosis—p21 endows malignant cells with a robust survival advantage under cytotoxic stress. Therapeutic interventions that disrupt p21’s survival-promoting functions, while sparing its tumor-suppressive checkpoint roles, could unlock new strategies in cancer treatment. This integrated role, which positions p21 as a critical determinant of therapeutic resistance by concurrently blocking multiple death pathways, see Fig. 2 and Table 1 for a summary of this integrated model.

**Table 1:** p21 as master orchestrator of broad-spectrum resistance to regulated cell death (RCD)

RCD Pathway	Role of p21	Key mechanisms of inhibition	Context & Therapeutic Implication	References
Apoptosis	Potent Inhibitor	Cytoplasmic p21 directly binds and inhibits ASK1, JNK, and procaspase-3/8/10	Major driver of chemo- and radiotherapy resistance; enables survival and clonal evolution of DNA-damaged cells.	^30,31^
Autophagy	Context-dependent; predominantly suppressor in therapy-resistant cells.	• Nuclear p21 can induce autophagy genes (ULK1, LC3) → minor pro-autophagic effect in specific stress contexts • Dominant cytoplasmic p21 strongly suppresses autophagic flux by inhibiting the Akt–ROS–FoxO axis and blocking autophagosome formation.	In apoptosis-resistant tumors, suppression of autophagy (the backup death pathway) consolidates long-term survival under metabolic/genotoxic stress - a hallmark of the p21 resilience network.	^5,35,36^
Ferroptosis	Potent suppressor	(1) Nrf2-independent stabilisation of GPX4 via LUBAC (2) Nrf2-dependent ↑GSH synthesis and ↑SLC7A11 expression.	Core component of the unified p21–Nrf2–GSH resilience axis; confers resistance to ferroptosis inducers (erastin, RSL3, FINs).	^39,40^
Cuproptosis	Potent suppressor (proposed and mechanistically unified here).	p21 → Nrf2 activation → ↑GSH synthesis → direct Cu⁺ chelation and prevention of lipoylated-protein aggregation (mechanistically identical downstream effector as in ferroptosis).	Establishes a unified p21–Nrf2–GSH axis that simultaneously suppresses both ferroptosis and cuproptosis - a novel broad-spectrum resilience mechanism proposed and mechanistically unified for the first time in this work.	^44–47^
Unified p21 resilience network	Master orchestrator of broad-spectrum RCD resistance	Simultaneous suppression of apoptosis, autophagy, ferroptosis, and cuproptosis + induction of prolonged cell-cycle arrest/senescence.	Generates highly persistent, therapy-refractory cancer cell populations; represents a high-value therapeutic target for next-generation combination strategies.	This work.

This table summarises how p21 simultaneously suppresses multiple lethal pathways, thereby establishing a highly persistent, therapy-refractory cellular state.

## Conclusion and future perspectives

p21 has long been celebrated as a tumor suppressor for its role in checkpoint enforcement and senescence induction^49^. Yet, as summarized in Table 1, growing evidence redefines it as a context-dependent survival factor. By inhibiting apoptosis, autophagy, ferroptosis, and potentially cuproptosis, p21 equips cells with a formidable resistance network.

The implications are twofold. First, therapeutic strategies must account for p21’s duality—its beneficial role in maintaining genomic integrity versus its detrimental role in promoting treatment resistance. Second, emerging death modalities such as ferroptosis and cuproptosis cannot be fully understood without considering p21’s influence.

### Limitations of the work

This perspective presents a conceptual framework based on literature synthesis and proposes a unified p21–Nrf2–GSH axis for ferroptosis and cuproptosis resistance. While supported by converging evidence, some aspects (e.g., the necessity of Nrf2 for p21’s protective effects) remain hypothetical and await direct experimental validation (e.g., epistasis experiments, genetic knockouts under specific stresses). The scope is focused on apoptosis, autophagy, ferroptosis, and cuproptosis due to the strength of available direct evidence linking p21 to these pathways; other regulated cell death pathways (e.g., necroptosis, pyroptosis) may also intersect with p21 biology but are beyond the current scope of this perspective.

Key open questions include:

- Which post-translational modifications or protein–protein interactions dictate whether p21 promotes tumor suppression or cell survival?
- How does the p21-Nrf2-GSH axis integrate stress signals to provide a unified defense against both ferroptotic and cuproptotic insults, and can this axis be therapeutically targeted to disrupt multiple resistance mechanisms simultaneously?
- Can selective inhibition of p21’s survival-promoting functions sensitize resistant cancers while preserving its DNA repair capacity?
- Does p21 coordinate cross-regulation among multiple cell death pathways, and could this serve as a therapeutic vulnerability?
- How can bioinformatics, computational systems biology, and mathematical modeling approaches predict p21-dependent resistance mechanisms and guide rational drug design?

Collectively, these questions provide a clear and actionable experimental roadmap to validate the central hypothesis of this Perspective: that p21 is a master regulator of a coordinated cell death defense network. Addressing these questions will require integrative experimental and computational frameworks that combine molecular biology, omics data analysis, and predictive modeling. Ultimately, redefining p21 as a master switch—not merely for cell cycle arrest but for survival against multiple death programs—may transform our understanding of therapy resistance and open avenues to strategically flip this molecular switch against cancer.

## Data Availability

No datasets were generated or analysed during the current study.

## References

[1] El-Deiry, W. S. et al. WAF1/CIP1 Is Induced in p53-mediated G1 arrest and apoptosis1. *Cancer Res*. **54**, 1169–1174 (1994). 8118801

[2] Sancar, A., Lindsey-Boltz, L. A., Unsal-Kaçmaz, K. & Linn, S. Molecular mechanisms of mammalian DNA repair and the DNA damage checkpoints. *Annu Rev. Biochem* **73**, 39–85 (2004). 15189136 10.1146/annurev.biochem.73.011303.073723

[3] Karakostis, K. et al. A single synonymous mutation determines the phosphorylation and stability of the nascent protein. *J. Mol. Cell Biol.* **11**, 187–199 (2018). 10.1093/jmcb/mjy049 PMC6734142 30252118

[4] Gartel, A. L. & Tyner, A. L. The role of the cyclin-dependent kinase inhibitor p21 in apoptosis. *Mol. Cancer Ther.* **1**, 639–649 (2002). 12479224

[5] Manu, K. A., Cao, P. H. A., Chai, T. F., Casey, P. J. & Wang, M. p21cip1/waf1 coordinates autophagy, proliferation and apoptosis in response to metabolic stress. *Cancers (Basel)* **11**, 1112 (2019). 31382612 10.3390/cancers11081112 PMC6721591

[6] Dixon, S. J. et al. Ferroptosis: An iron-dependent form of nonapoptotic cell death. *Cell* **149**, 1060–1072 (2012). 22632970 10.1016/j.cell.2012.03.042 PMC3367386

[7] Wang, Y., Zhang, L. & Zhou, F. Cuproptosis: A new form of programmed cell death. *Cell Mol. Immunol.* **19**, 867–868 (2022). 35459854 10.1038/s41423-022-00866-1 PMC9338229

[8] Wang, H., Guo, M., Wei, H. & Chen, Y. Targeting p53 pathways: Mechanisms, structures, and advances in therapy. *Signal Transduct. Target Ther.* **8**, 92 (2023). 36859359 10.1038/s41392-023-01347-1 PMC9977964

[9] Liu, Y. & Gu, W. p53 in ferroptosis regulation: The new weapon for the old guardian. *Cell Death Differ.* **29**, 895–910 (2022). 35087226 10.1038/s41418-022-00943-y PMC9091200

[10] Tarangelo, A. et al. p53 suppresses metabolic stress-induced ferroptosis in cancer cells. *Cell Rep.* **22**, 569–575 (2018). 29346757 10.1016/j.celrep.2017.12.077 PMC5791910

[11] Xu, R., Wang, W. & Zhang, W. Ferroptosis and the bidirectional regulatory factor p53. *Cell Death Discov.* **9**, 197 (2023). 37386007 10.1038/s41420-023-01517-8 PMC10310766

[12] Berghe, T. V., Linkermann, A., Jouan-Lanhouet, S., Walczak, H. & Vandenabeele, P. Regulated necrosis: The expanding network of non-apoptotic cell death pathways. *Nat. Rev. Mol. Cell Biol.* **15**, 135–147 (2014). 24452471 10.1038/nrm3737

[13] Conrad, M., Angeli, J. P. F., Vandenabeele, P. & Stockwell, B. R. Regulated necrosis: Disease relevance and therapeutic opportunities. *Nat. Rev. Drug Discov.* **15**, 348–366 (2016). 26775689 10.1038/nrd.2015.6 PMC6531857

[14] Bergsbaken, T., Fink, S. L. & Cookson, B. T. Pyroptosis: Host cell death and inflammation. *Nat. Rev. Microbiol* **7**, 99–109 (2009). 19148178 10.1038/nrmicro2070 PMC2910423

[15] Abbas, T. & Dutta, A. p21 in cancer: Intricate networks and multiple activities. *Nat. Rev. Cancer* **9**, 400–414 (2009). 19440234 10.1038/nrc2657 PMC2722839

[16] Xiong, Y. et al. p21 is a universal inhibitor of cyclin kinases. *Nature* **366**, 701–704 (1993). 8259214 10.1038/366701a0

[17] Cayrol, C., Knibiehler, M. & Ducommun, B. p21 binding to PCNA causes G1 and G2 cell cycle arrest in p53-deficient cells. *Oncogene* **16**, 311–320 (1998). 9467956 10.1038/sj.onc.1201543

[18] Maya-Mendoza, A. et al. High speed of fork progression induces DNA replication stress and genomic instability. *Nature* **559**, 279–284 (2018). 29950726 10.1038/s41586-018-0261-5

[19] Mansilla, S. F., de la Vega, M. B., Calzetta, N. L., Siri, S. O. & Gottifredi, V. CDK-independent and PCNA-dependent functions of p21 in DNA replication. *Genes (Basel)* **11**, 593 (2020). 32481484 10.3390/genes11060593 PMC7349641

[20] Yan, J. et al. The role of p21 in cellular senescence and aging-related diseases. *Mol. Cells* **47**, 100113 (2024). 39304134 10.1016/j.mocell.2024.100113 PMC11564947

[21] Shtutman, M., Chang, B.-D., Schools, G. P. & Broude, E. V. Cellular model of p21-induced senescence. *Methods Mol. Biol.* **1534**, 31–39 (2017). 27812865 10.1007/978-1-4939-6670-7_3 PMC6764449

[22] de Carné Trécesson, S. et al. Escape from p21-mediated oncogene-induced senescence leads to cell dedifferentiation and dependence on anti-apoptotic Bcl-xL and MCL1 proteins*. *J. Biol. Chem.* **286**, 12825–12838 (2011). 21292770 10.1074/jbc.M110.186437 PMC3075630

[23] Xiao, S. et al. Cellular senescence: a double-edged sword in cancer therapy. *Front Oncol.* **13**, 1189015 (2023). 37771436 10.3389/fonc.2023.1189015 PMC10522834

[24] Pérez-Mancera, P. A., Young, A. R. J. & Narita, M. Inside and out: the activities of senescence in cancer. *Nat. Rev. Cancer* **14**, 547–558 (2014). 25030953 10.1038/nrc3773

[25] Klepacki, H., Kowalczuk, K., Łepkowska, N. & Hermanowicz, J. M. Molecular regulation of SASP in cellular senescence: Therapeutic implications and translational challenges. *Cells* **14**, 942 (2025). 40643463 10.3390/cells14130942 PMC12248485

[26] Dong, Z. et al. Cellular senescence and SASP in tumor progression and therapeutic opportunities. *Mol. Cancer* **23**, 181 (2024). 39217404 10.1186/s12943-024-02096-7 PMC11365203

[27] Wang, B. et al. Intermittent clearance of p21-highly-expressing cells extends lifespan and confers sustained benefits to health and physical function. *Cell Metab.* **36**, 1795–1805.e6 (2024). 39111286 10.1016/j.cmet.2024.07.006 PMC11315361

[28] Galanos, P. et al. Chronic p53-independent p21 expression causes genomic instability by deregulating replication licensing. *Nat. Cell Biol.* **18**, 777–789 (2016). 27323328 10.1038/ncb3378 PMC6535144

[29] Kreis, N.-N., Louwen, F. & Yuan, J. Less understood issues: p21Cip1 in mitosis and its therapeutic potential. *Oncogene* **34**, 1758–1767 (2015). 24858045 10.1038/onc.2014.133

[30] Zhan, J. et al. Negative regulation of ASK1 by p21Cip1 involves a small domain that includes serine 98 that is phosphorylated by ASK1 in vivo. *Mol. Cell Biol.* **27**, 3530–3541 (2007). 17325029 10.1128/MCB.00086-06 PMC1899956

[31] Asada, M. et al. Apoptosis inhibitory activity of cytoplasmic p21Cip1/WAF1 in monocytic differentiation. *EMBO J.* **18**, 1223–1234 (1999). 10064589 10.1093/emboj/18.5.1223 PMC1171213

[32] El-Deiry, W. S. p21(WAF1) mediates cell cycle inhibition, relevant to cancer suppression and therapy. *Cancer Res.* **76**, 5189–5191 (2016). 27635040 10.1158/0008-5472.CAN-16-2055 PMC5028108

[33] Zhou, B. P. et al. Cytoplasmic localization of p21Cip1/WAF1 by Akt-induced phosphorylation in HER-2/neu-overexpressing cells. *Nat. Cell Biol.* **3**, 245–252 (2001). 11231573 10.1038/35060032

[34] Vincent, A. J. et al. Cytoplasmic translocation of p21 mediates NUPR1-induced chemoresistance. *FEBS Lett.* **586**, 3429–3434 (2012). 22858377 10.1016/j.febslet.2012.07.063

[35] Maheshwari, M. et al. Inhibition of p21 activates Akt kinase to trigger ROS-induced autophagy and impacts on tumor growth rate. *Cell Death Dis.* **13**, 1045 (2022). 36522339 10.1038/s41419-022-05486-1 PMC9755229

[36] Huang, S. et al. Autophagy is involved in the protective effect of p21 on LPS-induced cardiac dysfunction. *Cell Death Dis.* **11**, 554 (2020). 32694519 10.1038/s41419-020-02765-7 PMC7374585

[37] Fujiwara, K. et al. Pivotal role of the cyclin-dependent kinase inhibitor p21WAF1/CIP1 in apoptosis and autophagy. *J. Biol. Chem.* **283**, 388–397 (2008). 17959603 10.1074/jbc.M611043200

[38] White, E. & DiPaola, R. S. The double-edged sword of autophagy modulation in cancer. *Clin. Cancer Res* **15**, 5308–5316 (2009). 19706824 10.1158/1078-0432.CCR-07-5023 PMC2737083

[39] Venkatesh, D., Stockwell, B. R. & Prives, C. p21 can be a barrier to ferroptosis independent of p53. *Aging (Albany NY)* **12**, 17800–17814 (2020). 32979260 10.18632/aging.103961 PMC7585094

[40] Yang, Y. et al. FTO ameliorates doxorubicin-induced cardiotoxicity by inhibiting ferroptosis via P53–P21/Nrf2 activation in a HuR-dependent m6A manner. *Redox Biol.* **70**, 103067 (2024). 38316068 10.1016/j.redox.2024.103067 PMC10862061

[41] Zheng, Z. et al. P21 resists ferroptosis in osteoarthritic chondrocytes by regulating GPX4 protein stability. *Free Radic. Biol. Med.* **212**, 336–348 (2024). 38176476 10.1016/j.freeradbiomed.2023.12.047

[42] Tang, D., Chen, X. & Kroemer, G. Cuproptosis: A copper-triggered modality of mitochondrial cell death. *Cell Res* **32**, 417–418 (2022). 35354936 10.1038/s41422-022-00653-7 PMC9061796

[43] Tsvetkov, P. et al. Copper induces cell death by targeting lipoylated TCA cycle proteins. *Science* **375**, 1254–1261 (2022). 35298263 10.1126/science.abf0529 PMC9273333

[44] Zhang, H. et al. Self-boosting cuproptosis-based synergistic antitumor therapy by GSH-enhanced cocatalysis and copper efflux inhibition. *ACS Appl. Nano Mater.* **7**, 19341–19354 (2024).

[45] Liu, S. et al. Nanoenhanced-cuproptosis results from the synergy of calcium overload and GSH depletion with the increasing of intracellular Ca/Mn/Cu Ions. *Adv. Sci.* **12**, 2412067 (2025). 10.1002/advs.202412067 PMC11967785 39928524

[46] Liu, J. et al. NFE2L2 and SLC25A39 drive cuproptosis resistance through GSH metabolism. *Sci. Rep.* **14**, 29579 (2024). 39609608 10.1038/s41598-024-81317-x PMC11605005

[47] Chen, W. et al. Direct Interaction between Nrf2 and p21Cip1/WAF1 upregulates the Nrf2-mediated antioxidant response. *Mol. Cell* **34**, 663–673 (2009). 19560419 10.1016/j.molcel.2009.04.029 PMC2714804

[48] Hanahan, D. Hallmarks of cancer: New dimensions. *Cancer Discov.* **12**, 31–46 (2022). 35022204 10.1158/2159-8290.CD-21-1059

[49] Gorgoulis, V. et al. Cellular senescence: Defining a path forward. *Cell* **179**, 813–827 (2019). 31675495 10.1016/j.cell.2019.10.005

